# Effect of silver nanoparticles on human mesenchymal stem cell differentiation

**DOI:** 10.3762/bjnano.5.214

**Published:** 2014-11-10

**Authors:** Christina Sengstock, Jörg Diendorf, Matthias Epple, Thomas A Schildhauer, Manfred Köller

**Affiliations:** 1 Bergmannsheil University Hospital/Surgical Research, Ruhr-University Bochum, Bürkle-de-la-Camp-Platz 1, 44789 Bochum, Germany; 2 Inorganic Chemistry and Center for Nanointegration Duisburg-Essen (CeNIDE), University of Duisburg-Essen, Universitaetsstr. 5-7, 45117 Essen, Germany

**Keywords:** differentiation, differentiation marker, mesenchymal stem cells, nanoparticles, silver

## Abstract

**Background:** Silver nanoparticles (Ag-NP) are one of the fastest growing products in nano-medicine due to their enhanced antibacterial activity at the nanoscale level. In biomedicine, hundreds of products have been coated with Ag-NP. For example, various medical devices include silver, such as surgical instruments, bone implants and wound dressings. After the degradation of these materials, or depending on the coating technique, silver in nanoparticle or ion form can be released and may come into close contact with tissues and cells. Despite incorporation of Ag-NP as an antibacterial agent in different products, the toxicological and biological effects of silver in the human body after long-term and low-concentration exposure are not well understood. In the current study, we investigated the effects of both ionic and nanoparticulate silver on the differentiation of human mesenchymal stem cells (hMSCs) into adipogenic, osteogenic and chondrogenic lineages and on the secretion of the respective differentiation markers adiponectin, osteocalcin and aggrecan.

**Results:** As shown through laser scanning microscopy, Ag-NP with a size of 80 nm (hydrodynamic diameter) were taken up into hMSCs as nanoparticulate material. After 24 h of incubation, these Ag-NP were mainly found in the endo-lysosomal cell compartment as agglomerated material. Cytotoxicity was observed for differentiated or undifferentiated hMSCs treated with high silver concentrations (≥20 µg·mL^−1^ Ag-NP; ≥1.5 µg·mL^−1^ Ag^+^ ions) but not with low-concentration treatments (≤10 µg·mL^−1^ Ag-NP; ≤1.0 µg·mL^−1^ Ag^+^ ions). Subtoxic concentrations of Ag-NP and Ag^+^ ions impaired the adipogenic and osteogenic differentiation of hMSCs in a concentration-dependent manner, whereas chondrogenic differentiation was unaffected after 21 d of incubation. In contrast to aggrecan, the inhibitory effect of adipogenic and osteogenic differentiation was confirmed by a decrease in the secretion of specific biomarkers, including adiponectin (adipocytes) and osteocalcin (osteoblasts).

**Conclusion:** Aside from the well-studied antibacterial effect of silver, little is known about the influence of nano-silver on cell differentiation processes. Our results demonstrate that ionic or nanoparticulate silver attenuates the adipogenic and osteogenic differentiation of hMSCs even at non-toxic concentrations. Therefore, more studies are needed to investigate the effects of silver species on cells at low concentrations during long-term treatment.

## Introduction

Novel nanomaterials are being developed to enhance the diagnoses and treatment of diseases through the improved delivery of drugs, biopharmaceutical molecules and imaging agents to target cells at the sites of disease as well as through the surface treatment of biomaterials, such as implants. Ag-NP have a high degree of commercialization among current nanomaterials mainly due to their well-known antiseptic activities [1]. Presently, there are over 600 commercialized products on the market made from engineered nanomaterials, and Ag-NP are contained in approximately 250 of these products [2–3]. In the medical sector, various Ag nanomaterials have been used in numerous devices and products, such as silver sulfadiazine in the treatment of burns to reduce skin infections. Furthermore, silver has been used to coat a variety of different surfaces, such as catheters [4–7], or it has been incorporated into a hydrogel network for wound healing [8]. In our previous studies on the biological effects of Ag-NP (PVP-coated, 80 nm) on human mesenchymal stem cells (hMSCs), we have shown that cell activation could occur at elevated but non-toxic silver concentrations [9–10]. In addition, we have shown that hMSCs are able to ingest Ag-NP through clathrin-dependent endocytosis and by macropinocytosis and that silver agglomerates were formed in the cytoplasm following the uptake of these nanoparticles [11]. There is a general consensus that dissolved silver ions are responsible for the majority of the biological effects on various cells and that the generation of reactive oxygen species is involved in the silver-induced cell response [9,12–16].

Previously, we have shown that silver ions are more toxic to hMSCs than Ag-NP (in terms of the absolute concentration of silver) [9–10]. This effect is approximately three times higher for silver ions than for Ag-NP; however, the biological effects induced by both nanoparticulate and ionic silver occurred in the same respective concentration ranges for eukaryotic cells and microorganisms [17–19]. We and others have studied the mechanisms underlying silver ion release from nanoparticles [20–21]. The release of silver ions seems to involve a cooperative oxidation process that requires both dissolved dioxygen and protons. The ion release rates increase with temperature in the range of 0–37 °C and decrease with increasing pH [21–22]. However, the presence of ligands (such as SO_4_^2−^, S^2−^) in the microenvironment considerably decreased the adverse effects of silver ions and silver nanoparticles, indicating that these ligands bind silver [18,23]. Today, Ag-NP are increasingly used because particles with sizes in the range of a few nanometers lead to a dramatic increase in the surface area/mass ratio in contrast to micrometer-sized particles. Such an enlargement of the reactive surface area will lead to the effective release of silver ions (Ag^+^) in parallel with low total silver concentrations, resulting in an increased release effect with respect to the applied mass of silver [23–24].

Because of the high differentiating capacity of hMSCs they are an optimal cell model to analyze the possible influence of silver nanoparticles on cell differentiation. MSCs are neither transformed nor immortalized cells, rather, they represent primary pre-tissue cells. They can therefore be cultured for weeks without cell passage, which is important for long-term studies [25]. Furthermore, MSCs contribute to the regeneration and repair of mesenchymal tissues such as bone, cartilage, muscle, ligaments, tendons, adipose tissue and stroma [26].

Ag-NP and Ag^+^ ions have been reported to bind rapidly to biomolecules, such as DNA [27], negatively charged cell-wall components and the sulfhydryl groups of metabolic enzymes [7,28–29], which results in the inhibition of DNA-replication, an increase in membrane permeability and the disturbance of different metabolic pathways [30]. In contrast to the vast number of toxicological and microbiological studies [7,9,21], only a few studies have investigated whether the differentiation potential of hMSCs was maintained after the uptake of different nanoparticles [31–33].

Therefore, the aim of this study was to investigate the cellular uptake of nano-silver by hMSCs and the influence of nanoparticulate or ionic silver on the viability and differentiation potential of these cells. The viability and adipogenic, osteogenic and chondrogenic differentiation potential were examined qualitatively and quantitatively through light and fluorescence microscopy, photometry and by analyzing the secretion of typical biomarkers.

## Results

### Uptake and intracellular distribution of nano-silver in hMSCs

**Figure 1:**
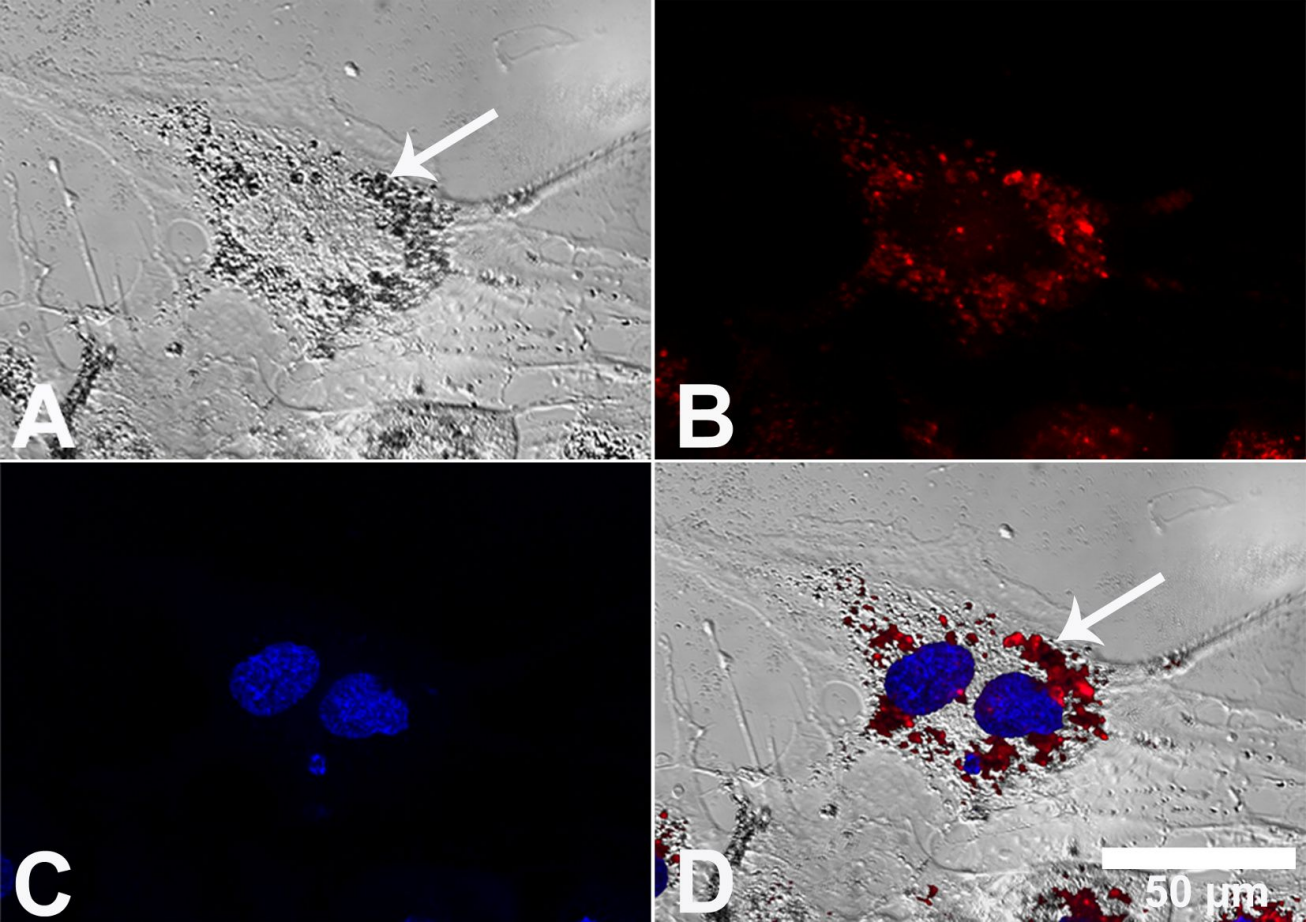
Localization of Ag-NP agglomerates in hMSCs. Representative light micrographs after digital contrast enhancement (DCE filter) (A), laser scanning micrographs (B,C) and combinations of both (D) are shown. The white arrow denotes the intracellular accumulation of silver particles inside the endo-lysosomes (A,D). The blue fluorescence of Hoechst 33342 and the red fluorescence of LysoTracker Red DND 99, which were used as probes of the cell nucleus (C) and endo-lysosomes (B), respectively, are shown.

Human MSCs were cultured in the presence of 20 µg·mL^−1^ Ag-NP at 37 °C for 24 h under cell culture conditions, and the cell nucleus and endo-lysosomes were labeled with specific organelle markers. Laser scanning microscopy and phase-contrast microscopy were performed in parallel on identical cell areas (Figure 1A). In cells cultured in the presence of Ag-NPs, agglomerated nanoparticles were visible in a region close to the cell nucleus but not in the cell culture medium outside the cells. As shown in Figure 1D, silver agglomerates were mainly found associated with the endo-lysosomal areas (white arrow denotes silver agglomerates) but not inside the nucleus (Figure 1A and Figure 1D). A similar culture of hMSCs, in the presence of silver acetate (data not shown), did not reveal any silver agglomerate formation.

### Adipogenic differentiation of hMSCs after silver exposure

**Figure 2:**
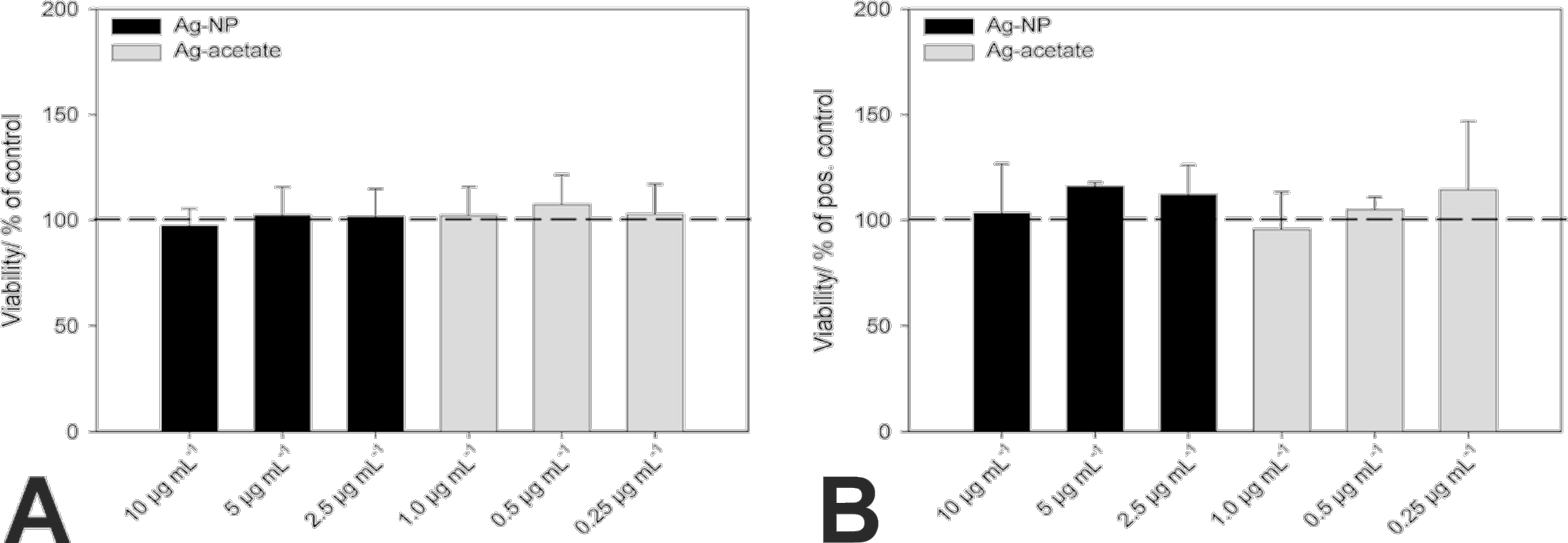
Influence of different Ag-NP/Ag^+^ ion concentrations on the viability of undifferentiated hMSCs (A) and adipogenic-differentiated hMSCs (B). After 14 d of incubation, viable cells were stained with calcein-AM (green fluorescence) and quantified by using digital image processing (phase analysis). The data are expressed as the mean ± SD (*n* = 3 independent experiments) given as the percentage of viable cells cultured in the presence of RPMI/FCS (A) or in the presence of adipogenic-differentiation medium (B).

In order to analyze the influence of nano-silver on the viability of undifferentiated cells (as a negative control) and on adipogenic-differentiated stem cells, hMSCs were cultured in the presence of different concentrations of Ag-NP/Ag^+^ ions for 24 h. The medium that contained the particles was subsequently removed and exchanged with pure RPMI/FCS (for undifferentiated cells) or adipogenic-differentiation medium, and the hMSCs were further cultivated for 14 d. As shown in Figure 2A, the silver concentrations used are not toxic to undifferentiated hMSCs after 14 d of incubation, which confirms our earlier reports [9–10,17,19]. Similarly, the addition of Ag-NP (black bars) or Ag^+^ ions (grey bars) did not decrease the viability of adipogenic-differentiated hMSCs at the concentrations used (Figure 2B).

**Figure 3:**
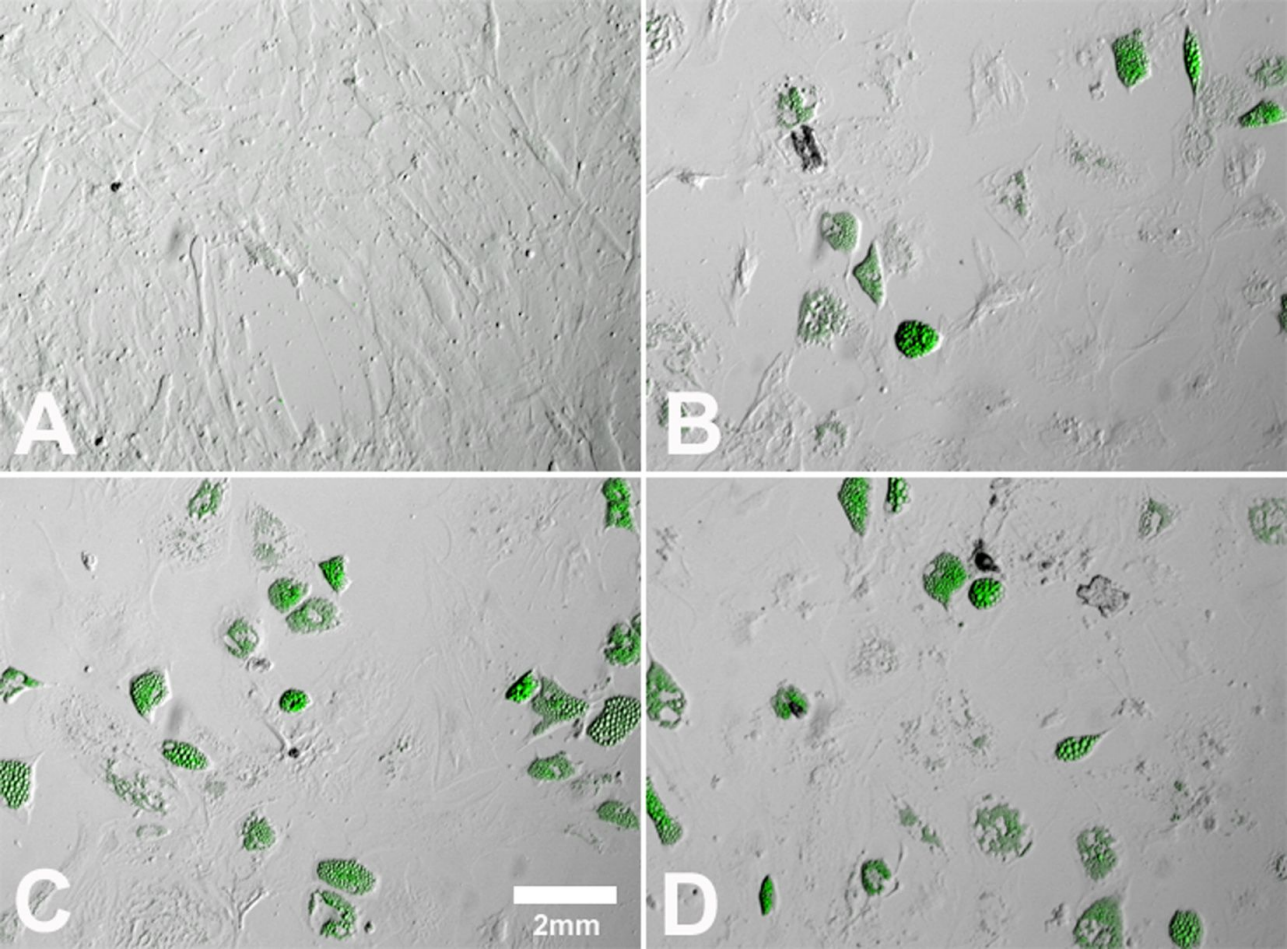
Influence of Ag-NP/ Ag^+^ ions on the adipogenic differentiation of hMSCs. After 14 d of cell culture (bright-field and fluorescence images), Bodipy^493/503^ staining was used to visualize lipid vacuoles in cells cultured under adipogenic conditions. hMSCs incubated in the presence of RPMI/10% FCS served as a negative control (A). hMSCs incubated in the presence of adipogenic-differentiation media served as a positive control (C). Cells were incubated with 10 µg·mL^−1^ Ag-NP (B) or with 1.0 µg·mL^−1^ Ag^+^ ions (D) for 24 h and were subsequently incubated with pure adipogenic-differentiation media for further 14 d.

To confirm adipogenic differentiation, the lipid content of the cells was visualized by using oil red O or Bodipy^493/503^ staining. As shown in Figure 3C, hMSCs differentiated into adipocytes in the presence of adipogenic-differentiation media (positive control; Figure 3C), in contrast to cells that were cultivated in the presence of RPMI/FCS (negative control; Figure 3A). Differentiated hMSCs changed their morphology from a fibroblast-like form (Figure 3A) to a spherical one with intracellular lipid droplets (Figure 3C). Cells that were additionally treated with 10 µg·mL^−1^ Ag-NP (Figure 3B) or 1.0 µg·mL^−1^ Ag^+^ ions (Figure 3D) revealed no significant morphological changes compared with cells cultured without silver (Figure 3C). However, a large decrease in the number of formed lipid droplets was observed in the presence of a high concentration of Ag-NP or Ag^+^ ions (Figure 3B and Figure 3D).

**Figure 4:**
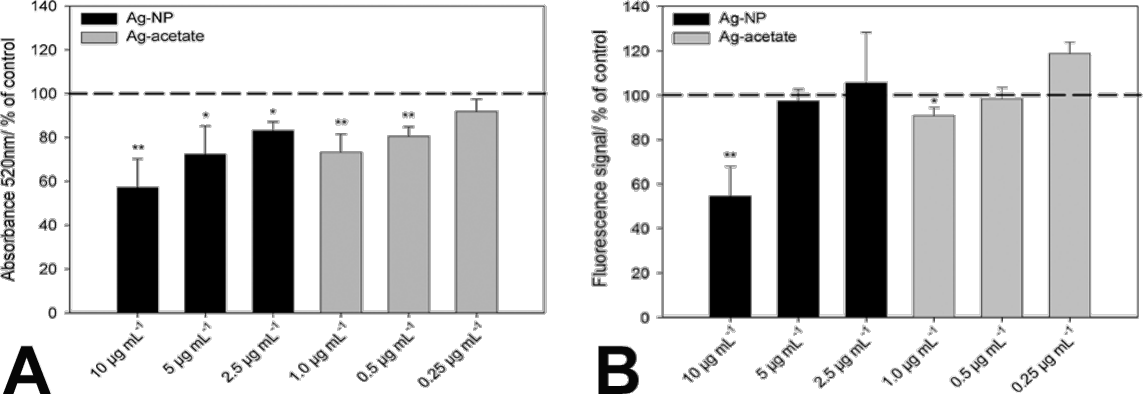
Influence of Ag-NP (black bars) or Ag^+^ ions (grey bars) on the adipogenic differentiation of hMSCs after 14 d of incubation. Quantitative analyses of lipid droplet accumulation were performed by measuring the optical density (520 nm) of extracted oil red-stained lipid droplets (A) or by phase analysis of lipid droplets stained with Bodipy^493/503^ (B). The data are expressed as the mean ± SD (*n* = 3 independent experiments) given as the percentage of cells cultured under adipogenic conditions in the absence of silver. The asterisks (*) indicate significant differences in comparison to the control (*p < 0.05;**p < 0.005).

The adipogenic differentiation of hMSCs was quantified by the optical density (520 nm) of the extracted oil red-stained lipid droplets and by phase analysis of cells stained with Bodipy^493/503^. As shown in Figure 4, the quantification of oil red (Figure 4A) and Bodipy^493/503^ (Figure 4B) revealed a decrease in lipid vacuoles with increasing silver concentrations. This decrease was significant at the applied concentrations of 10 µg·mL^−1^ for Ag-NP (black bars) or 1.0 µg·mL^−1^ for Ag^+^ ions (grey bars). The differences between oil red extraction and phase analysis after Bodipy^493/503^ staining may be due to the different extraction of oil red in the presence of silver.

**Figure 5:**
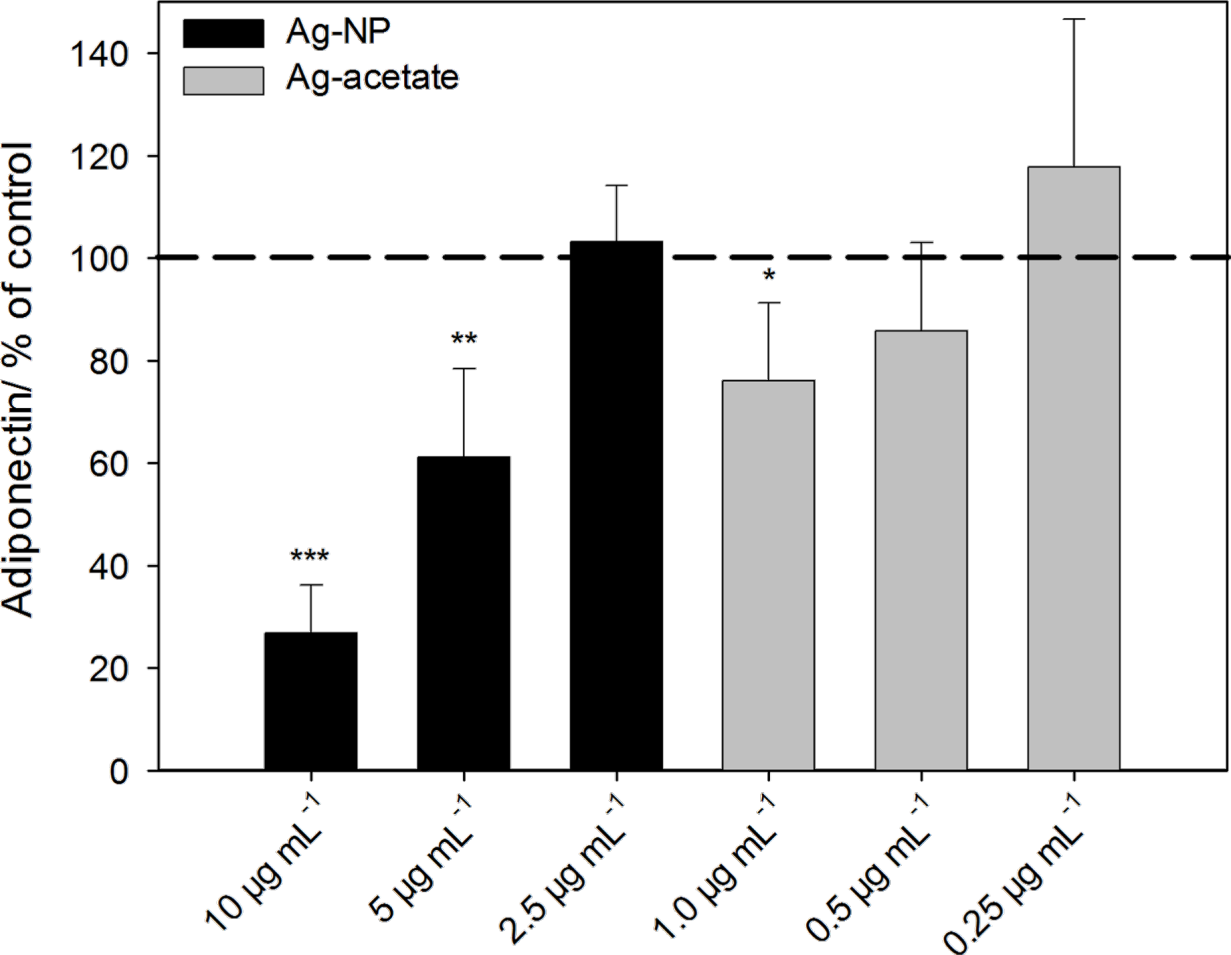
Release of the adipogenic differentiation marker adiponectin after hMSCs were incubated with Ag-NP/Ag^+^ ions. After incubation with different concentrations of Ag-NP/Ag^+^ ions for 24 h, the silver-treated cells were washed, and particle-free medium (adipogenic-differentiation medium) was added. After 14 d of incubation, the amount of released adiponectin was measured. The data are expressed as the mean ± SD (*n* = 7 independent experiments), given as the percentage of the control (Adiponectin 191 ± 13 ng·mL^−1^; cells cultured without silver, dashed line). The asterisks (*) indicate significant differences with respect to the control (*p < 0.05, **p < 0.01, ***p < 0.001).

To further investigate the effect of Ag-NP on the adipogenic differentiation of hMSCs, the expression of adiponectin was analyzed by using ELISA. Adiponectin is specifically secreted by adipose tissue and can be used as a marker for the adipogenic differentiation of MSCs. As shown in Figure 5, the release of adiponectin decreased significantly at Ag-NP concentrations of 5 µg·mL^−1^ and 10 µg·mL^−1^ (black bars), in contrast to untreated control cells (dashed line). Similar results were observed in cells treated with 1 µg·mL^−1^ Ag^+^ ions (grey bars).

### Osteogenic differentiation of hMSCs after silver exposure

The viability of osteogenic-differentiated hMSCs was assessed as described in the Experimental section under quantitative cell differentiation. Undifferentiated cells were exposed to Ag-NP/Ag^+^ ions for 24 h and then cultivated for 21 d in the presence of RPMI/FCS without extracellular silver. No significant decrease in viability was observed. Similar results were observed for hMSCs that were cultured in the presence of osteogenic-differentiation media after incubation with Ag-NP/Ag^+^ ions (data not shown).

**Figure 6:**
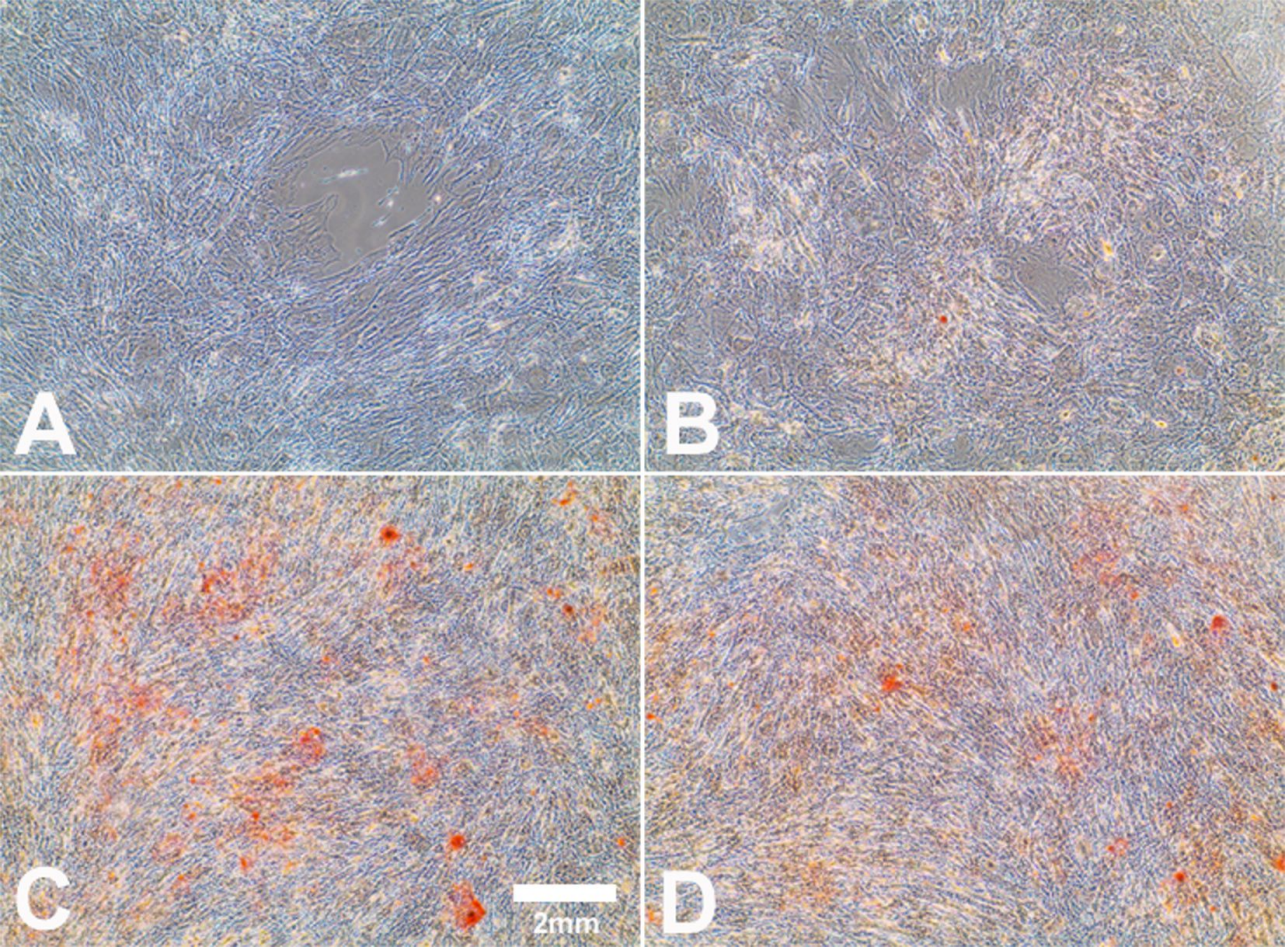
Influence of Ag-NP/ Ag^+^ ions on hMSCs during osteogenic differentiation. After 21 d of cell culture (bright-field images), staining with alizarin red S was used to visualize calcium accretion in cells cultured under osteogenic conditions. hMSCs incubated in the presence of osteogenic-differentiation media served as the positive control (C). hMSCs incubated in the presence of RPMI/FCS served as the negative control (A). hMSCs incubated with 10 µg·mL^−1^ Ag-NP (B) or with 1.0 µg·mL^−1^ Ag^+^ ions (D) for 24 h, and followed by osteogenic-differentiation media for a further 21 d.

The influence of Ag-NP/Ag^+^ ions on the osteogenic differentiation of hMSCs was investigated after a period of 21 d. To confirm the differentiation of the Ag-NP/Ag^+^ ion-treated hMSCs into osteoblasts, alizarin red S staining was carried out to verify the mineralization of the cells. hMSCs exposed to 10 µg·mL^−1^ Ag-NP displayed no distinct morphological changes, but a decrease in the differentiation of hMSCs (Figure 6B) in contrast to cells cultured without silver (Figure 6C) was observed. In the presence of Ag^+^ ions, no significant change in calcium accretion was measured (Figure 6D) in contrast to the positive control (Figure 6C).

**Figure 7:**
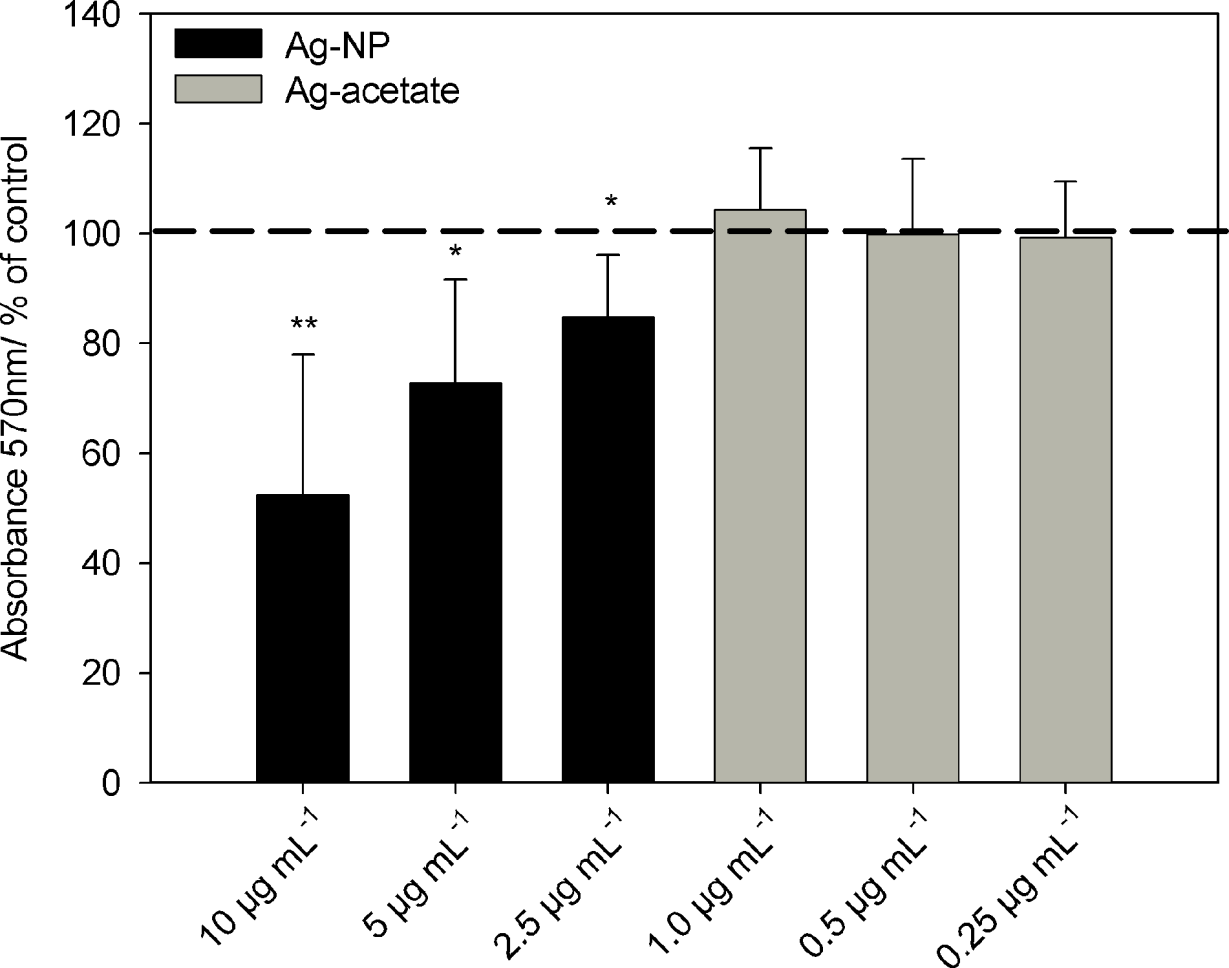
Influence of Ag-NP/Ag^+^ ions on the osteogenic differentiation of hMSCs after 21 d of incubation. Quantitative analyses of mineralization were performed by measuring the optical density (570 nm) of extracted alizarin red S. The data are expressed as the mean ± SD (*n* = 3 independent experiments), given as the percentage of cells cultured under osteogenic conditions in the absence of silver. The asterisks (*) indicate significant differences with respect to to the control (*p < 0.05; **p < 0.005).

The microscopic data were confirmed by quantitative analyses of osteogenic differentiation using cetylpyridinium chloride after the extraction of alizarin red. As shown in Figure 7, there was a significant change in calcium accretion after 21 d in the presence of Ag-NP. However, exposure to Ag^+^ ions at any of the concentrations used did not influence the osteogenic differentiation potential under these conditions (Figure 7; grey bars).

**Figure 8:**
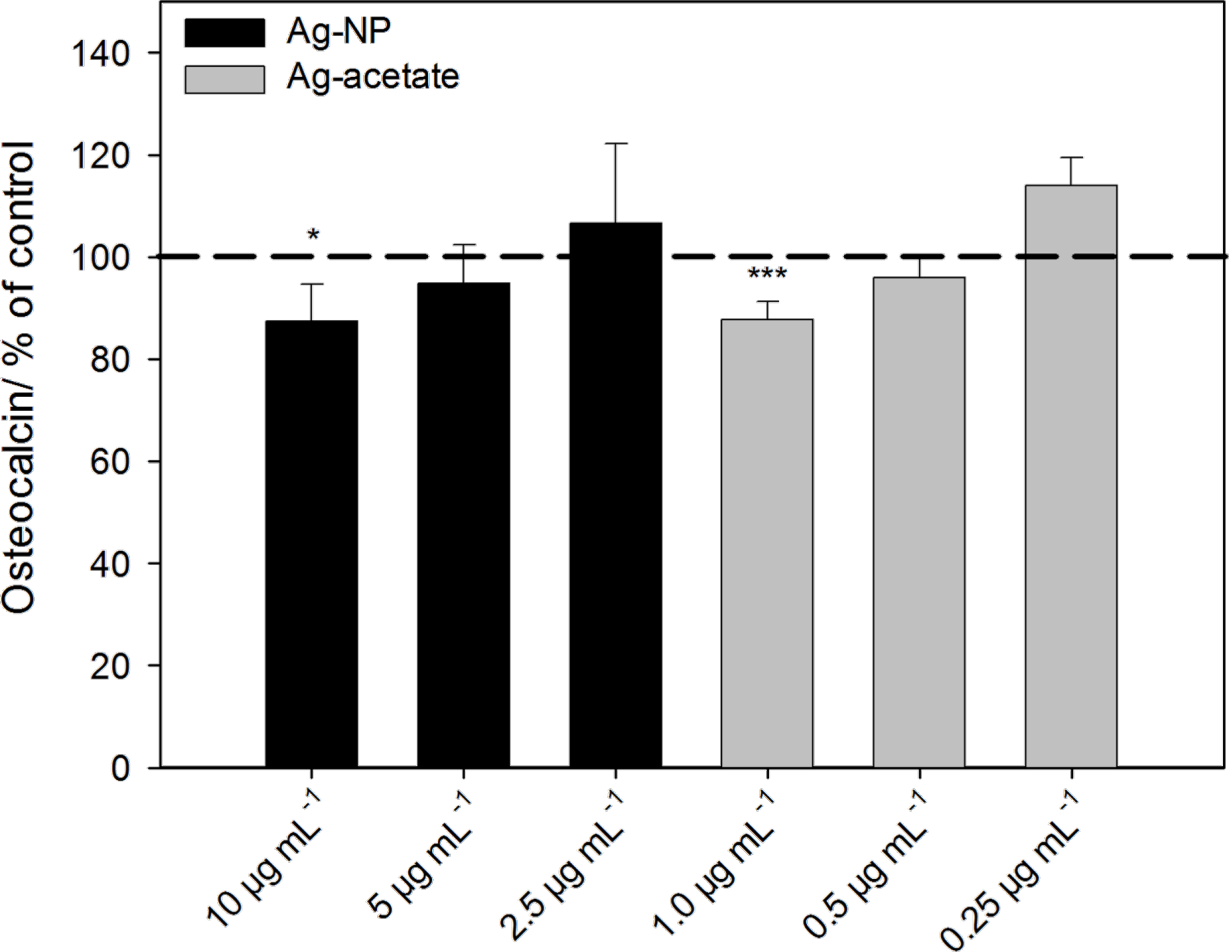
Release of the osteogenic differentiation marker osteocalcin after incubation of hMSCs with Ag-NP/Ag^+^ ions. The data are expressed as the mean ± SD (*n* = 7 independent experiments), given as the percent of the control (osteocalcin 15.6 ± 6 ng·mL^−1^; cells cultured without silver, dashed line). The asterisks (*) indicate significant differences with respect to the control (*p < 0.05, ***p < 0.001).

Osteocalcin was measured by performing ELISA using the supernatants of cells that were differentiated into the osteogenic lineage. Osteocalcin is a suitable biomarker for osteogenic differentiation when differentiation is analyzed after a prolonged period of three weeks under differentiating conditions. Although alkaline phosphatase activity occurs earlier, osteocalcin expression is detectable in MSCs after the first week of osteogenic induction and remains detectable from then on [34]. After 21 d of osteogenic differentiation the expression of osteocalcin was significantly decreased at 10 µg·mL^−1^ Ag-NP or 1 µg·mL^−1^ Ag^+^ ions (Figure 8).

### Chondrogenic differentiation of hMSCs after silver exposure

The viability of chondrogenic-differentiated cells was analyzed by calcein-AM staining and revealed viable cells. However, a quantitative analysis was not possible due to the typical cell conversion as a pellet. Silver concentration-dependent differences in the conversion process or in the pellet size were not observed.

**Figure 9:**
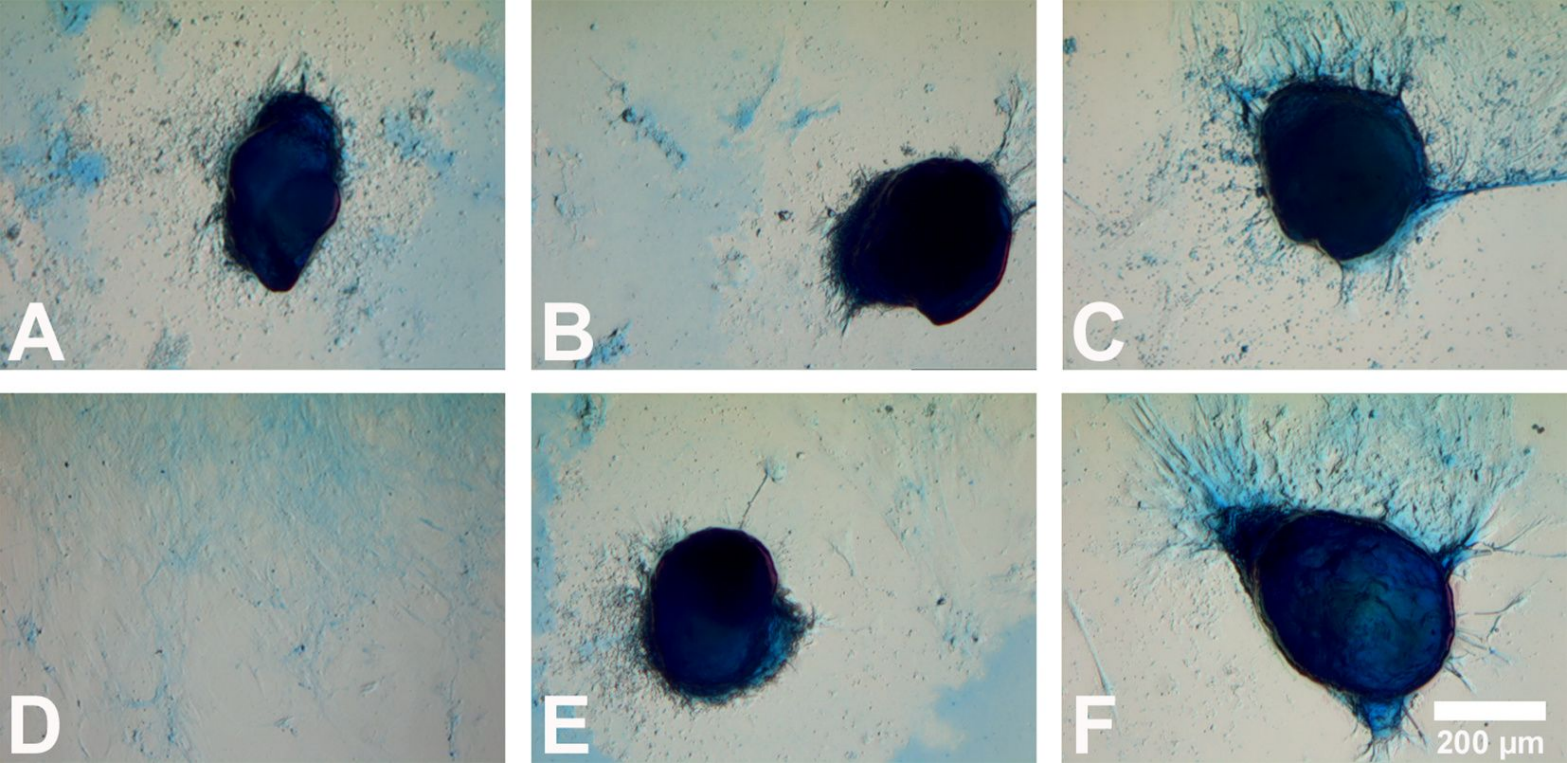
Influence of Ag-NP/Ag^+^ ions on the chondrogenic differentiation of hMSCs. After 21 d of cell culture under chondrogenic conditions (bright-field images), alcian blue staining was used to visualize polyanionic glycosaminoglycan chains of proteoglycans in the cultured cells. hMSCs incubated in the presence of chondrogenic-differentiation media served as the positive control (A). hMSCs incubated in the presence of RPMI/FCS served as the negative control (D). Cells were incubated with 10 µg·mL^−1^ (B) or with 5 µg·mL^−1^ (C) Ag-NP in the presence of chondrogenic differentiation media. In addition, cells were incubated with 1.0 µg·mL^−1^ (E) or 0.5 µg·mL^−1^ (F) Ag^+^ ions in the presence of chondrogenic differentiation media.

To investigate the effect of Ag-NP/Ag^+^ ions on the chondrogenic differentiation of hMSCs, alcian blue staining was used to visualize chondrocyte-typical proteoglycans. As shown in Figure 9, the potential for hMSCs to differentiate into chondrocytes was not influenced by the treatment with Ag-NP or Ag^+^ ions. No qualitative differences with respect to the intensity and distribution of alcian blue staining were found between cells that were cultured with or without silver. The size of the pellets (longest diameter) ranged from 200–900 µm regardless of the presence or absence of silver.

**Figure 10:**
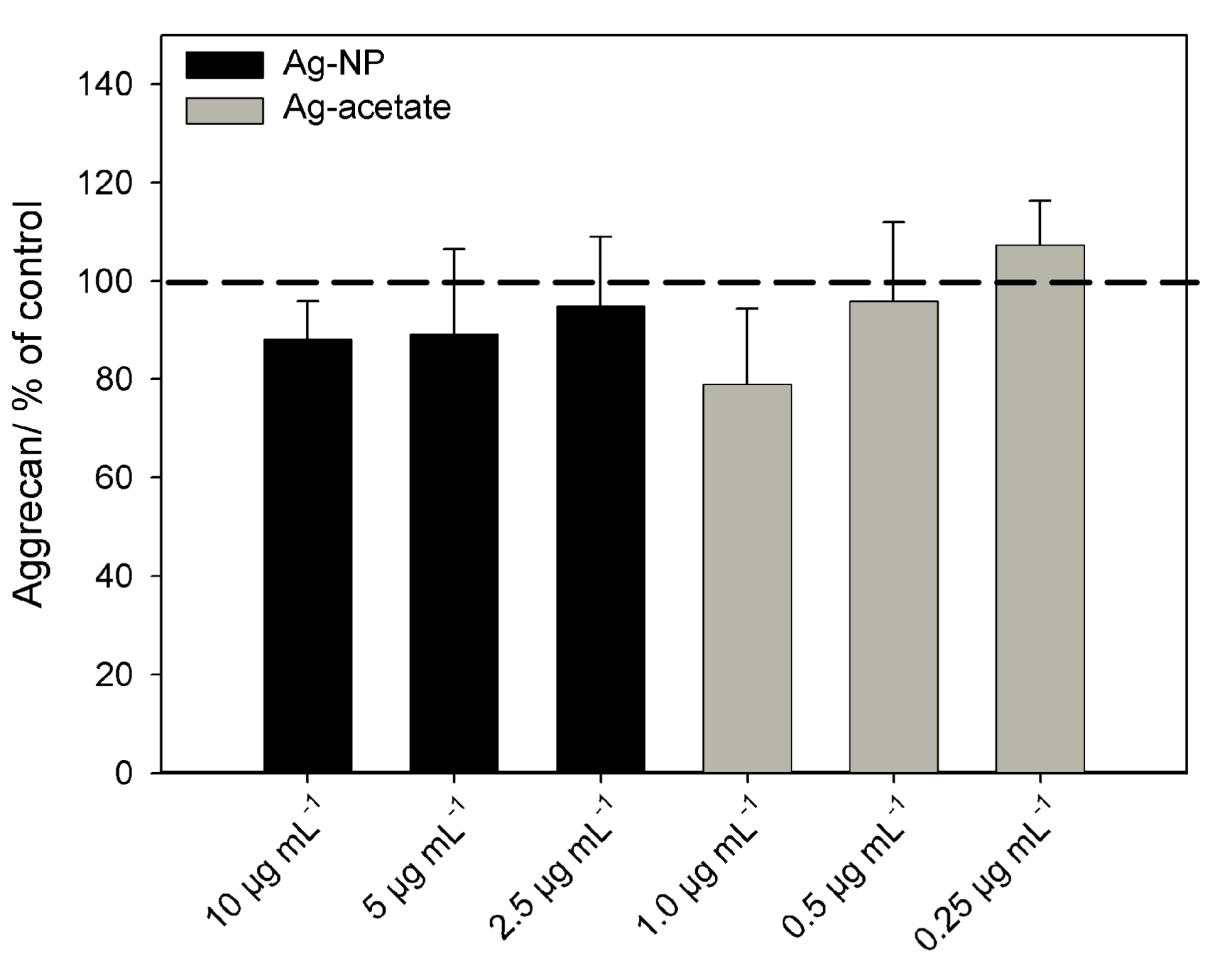
Release of the chondrogenic differentiation marker aggrecan after incubation of hMSCs with Ag-NP/Ag^+^ ions. The data are expressed as the mean ± SD (*n* = 7 independent experiments), given as the percentage of the control (aggrecan 5 ± 1.9 ng·mL^−1^; cells cultured without silver, dashed line).

In addition, the release of the chondrocyte biomarker aggrecan in the presence or absence of Ag-NP/Ag^+^ ions was measured by using ELISA (Figure 10). The structural proteoglycan aggrecan is found in the extracellular matrix of cartilage and is a suitable biomarker for chondrogenic differentiation of MSCs at late time points (at least three weeks), as in our experimental setup [35]. Aggrecan was not detectable in the supernatants of undifferentiated MSCs, but it was expressed in the supernatants of cells that were cultured in the presence of the chondrogenic-differentiation media. Similar to the alcian blue staining, there were no significant differences between cells that were treated with or without silver (ionic or particulate).

## Discussion

In the present study, we analyzed the effects of Ag-NP on hMSCs differentiation. We have shown that Ag-NP with a size of 80 nm do not enter the cell nucleus and that silver agglomerates appeared primarily within the endo-lysosomes after 24 h. Fröhlich et al. [36] have reported that access to other organelles depends on the particle size.

Similarly, as suggested by Berry et al., the uptake of nanoparticles is constrained by the dimensions of the nuclear pore because gold nanoparticles (Au-NP) with a size of 5 nm appeared in the nuclei of a human fibroblast cell line, whereas particles larger than 30 nm were retained in the cytoplasm [37]. In the present study, we used silver nanoparticles with a size of 80 nm, and thus, no silver agglomerates were found in the nucleus. Important aspects of the behavior of Ag-NP, such as cell toxicity or antimicrobial potency, are related to the reactivity of silver ions [38–41]. As we have shown previously, the rate and degree of the dissolution of Ag-NP depends on their surface functionalization, their concentration, the oxygen content and temperature [19,21]. Therefore, it has been suggested that Ag-NP act as a “Trojan horse” that enables the release of metal ions within cells [42–44]. In this context, it is important to analyze the influence of subtoxic concentrations of nano-silver on stem cell differentiation after long-term incubation. Human MSCs may come into close contact with nano-silver, e.g., after the implantation of an Ag-NP-coated implant [17,45]. To date, little is known about the influence of nanoparticles on stem cell differentiation. In this study, we have shown that the adipogenic and osteogenic differentiation of hMSCs was impaired at subtoxic concentrations of Ag-NP and Ag^+^ ions, whereas chondrogenic differentiation was not influenced by the presence of silver. Similar results were observed by Fan et al. when using gold nanoparticles [46]. These nanoparticles, with a size of 30 nm, led to decreases in the osteogenic and adipogenic differentiation capability of human bone MSCs. In addition, Kohl et al. reported that Au-NP led to a decrease in mitochondrial activity and inhibited lipid formation that depend on the concentration of the applied particles [47]. Therefore, the expression of adipogenic-specific genes and proteins is also expected to be attenuated in MSCs upon nanoparticle treatment [48]. This was confirmed in our study by the decreased secretion of specific biomarkers, including adiponectin (adipocytes) and osteocalcin (osteoblasts).

Liu et al. also used PCR to analyze osteocalcin expression in hMSCs at an early time-point (day 10) after osteogenic induction in the presence of Ag-NP [49]. The authors found no differences with respect to control cells without Ag-NP exposure, although a decrease in cell viability was also observed at Ag-NP concentrations ≥10 μg·mL^−1^. Pauksch et al. analyzed the expression of alkaline phosphatase in MSCs in the presence of Ag-NP (up to 1 μg·mL^−1^) after prolonged cell sulture (35 d). In contrast to the significant inhibition in cell proliferation observed at the highest concentration of Ag-NP, the authors found no influence on the activity of alkaline phosphatase [50].

Metal ions are generally assumed to have a significant effect on the activation of redox-sensitive transcription factors such as NF-κB or AP-1 [51]. These transcription factors are involved in inflammatory responses and are important for processes such as differentiation and cell growth [52]. Silver-mediated oxidative stress can lead to the nuclear translocation of NF-κB, which regulates pro- and anti-inflammatory genes [53–55]. For example, we previously demonstrated that Ag-NP-induced an activation of hMSCs and monocytes that was characterized by differential cytokine release (e.g., increased IL-8 or decreased IL-6 release), the increased expression of adhesion molecules such as CD54 and the enhanced generation of reactive oxygen species (ROS) [9–10]. As such, these observations indicated that the generation of ROS can be regarded as a common mechanism for silver-induced effects.

Other reports also showed that mechanical stress can inhibit adipogenesis, which is associated with the down-regulation of related marker genes [48,56–57]. However, whether the response is triggered by mechanical stress during membrane interaction and uptake or by the interaction of particles with intracellular signaling structures is still not clear. Studies specifically investigating the effect of silver on stem cell differentiation are rare [49–50,58]. Albers et al. have shown that Ag-NP with a size of 50 nm also led to a concentration-dependent decrease in murine osteogenic cell differentiation [59]. However, Samberg et al. reported no influence of Ag-NP (commercially available 10 nm and 20 nm particles) on adipose-derived stem cells based on photographic image analyses [60]. In contrast, we used bone-marrow-derived stem cells and additionally performed chondrogenic differentiation and quantitative analyses of histochemical staining, which might explain the differences in the obtained results. In the case of the chondrogenic differentiation, a quantitative analysis of alcian blue staining was not possible due to the 3-dimensional growth of chondrogenic-derived hMSCs. However, cell morphology and aggrecan release demonstrated no differences compared with cells cultured without silver. Similar results were obtained by Tautzenberger et al., who demonstrated that the chondrogenic differentiation of hMSCs was not influenced in the presence of polystyrene nanoparticles [31].

A major difficulty when comparing studies on the biological effects of nano-silver is the difference in the nature of the respective particle. In addition to the size, shape, or surface charge, the surface coating or functionalization used to stabilize the monodisperse nature is of critical importance. An important requirement is the agglomeration control because the agglomeration of particles might already occur within the incubation media [18,21]. Agglomerated nano-silver will follow different uptake mechanisms, if at all, and biological responses might occur at much higher total silver concentrations compared with monodispersed silver nanoparticles. In summary, Ag-NP exert multiple effects on cellular physiology and signal transduction, which can lead to alterations in gene expression. The adipogenic and osteogenic differentiation of hMSCs is clearly down-regulated even at subtoxic concentrations of Ag-NP.

## Conclusion

In conclusion, silver nanoparticles with a size of 80 nm (hydrodynamic diameter) were ingested into hMSCs as nanoparticulate material. After cellular uptake, these Ag-NP were mainly associated with the endo-lysosomal cell compartment and occurred as silver agglomerates within these organelles. Exposure of hMSCs to subtoxic concentrations of Ag-NP, as well as to Ag^+^ ions, during differentiation into adipogenic or osteogenic lineage resulted in a significant concentration-dependent decrease in differentiation capacity. Furthermore, the chondrogenic differentiation of hMSCs was not influenced by the presence of silver under these experimental conditions. In summary, the internalization of nano-silver into stem cells had a significant influence on diverse aspects of cellular functions. Therefore, more studies are needed to investigate the effects of nano-silver in directing stem cell behavior in order to predict the possible health risks.

## Experimental

### Synthesis of silver nanoparticles

Polyvinylpyrrolidone (PVP)-coated silver nanoparticles were synthesized by reduction with glucose in the presence of PVP as described previously [19,21]. The final silver concentration in all dispersions was determined by atomic absorption spectroscopy (AAS, Thermo Electron Corporation, M-Series). The hydrodynamic diameter and the zeta-potential of the dispersed particles were measured by dynamic light scattering (DLS) using a Malvern Zetasizer Nano ZS. The z-average value was used as the average particle diameter. The polydispersity index (PDI) was below 0.3 in all cases, indicating the absence of aggregates. Scanning electron microscopy (FEI Quanta 400 ESEM instrument) revealed a spherical shape of the Ag-NP used with a metallic core of 50 ± 20 nm. The hydrodynamic diameter of the nanoparticles was 80 nm as measured by DLS. Note that the hydrodynamic diameter includes the polymer layer and the hydration shell and is therefore always larger than the pure metal diameter of the silver core as determined by electron microscopy under high vacuum.

PVP (K30, Povidon 30; Fluka, molecular weight 40,000 g·mol^−1^), trisodium citrate dihydrate (Fluka, p.a.), silver nitrate (Fluka, p.a.), and D-(+)-glucose (Baker) were used. Ultrapure water was prepared with an ELGA Purelab ultra instrument. Ag-NP were stored under argon to prevent partial oxidative dissolution (which drastically influences nanoparticle toxicity) prior to cell culture experiments [19,21].

### Cell culture

Human mesenchymal stem cells (hMSCs, 3rd to 7th passage, Lonza, Walkersville Inc., MD, USA) were cultured in RPMI1640 cell culture medium (Life Technologies, Darmstadt, Germany) containing 10% fetal calf serum (FCS, Life Technologies) and L-glutamine (0.3 g·L^−1^, Life Technologies) while using 75 cm^2^ flasks (Falcon, Becton Dickinson GmbH, Heidelberg, Germany). Cells were maintained at 37 °C in a humidified 5% CO_2_ atmosphere. hMSCs were sub-cultivated every 7–14 d depending on cell proliferation. Adherent cells were washed with phosphate buffered saline solution (PBS, Life Technologies) and detached from the culture flasks by the addition of 0.2 mL·cm^−2^ 0.25% trypsin/0.05% ethylenediaminetetraacetic acid (EDTA, Sigma-Aldrich, Taufkirchen, Germany) for 5 min at 37 °C. Subsequently, the hMSCs were collected and washed twice with RPMI1640/10% FCS.

#### Determination of cellular Ag-NP uptake by laser scanning microscopy (LSM)

LSM was performed to demonstrate the occurrence of intracellular silver nanoparticles in hMSCs after incubation. Therefore, hMSCs were subconfluently grown on 2-well Lab-Tek^TM^ glass chamber slides (Thermo Fisher Scientific, Langenselbold, Germany) and subsequently washed and exposed to 20 µg·mL^−1^ Ag-NP for 24 h under cell culture conditions. Microscopic uptake studies require preferably high (but still non-toxic after 24 h) concentrations of Ag-NP to obtain an optimal read-out. After incubation, hMSCs were labeled with specific cell organelle fluorescent probes (Life Technologies). As a marker for late endosomes and lysosomes, the cells were incubated with 50 nM LysoTracker Red DND 99 in pure RPMI1640 for 30 min at 37 °C. To label the nucleus, the cells were incubated with 162 µM Hoechst 33342 in pure RPMI1640 for 5 min at 37 °C. After three rinses in RPMI1640, the cells were mounted on glass chamber slides. Images were taken with a laser scanning microscope (LSM 700; Zeiss) equipped with a 40× oil-immersion objective using Zeiss 2010 software.

#### Induction of hMSCs differentiation

For adipogenic and osteogenic differentiation, hMSCs were seeded subconfluently into 24-well tissue culture plates (Falcon, Becton Dickinson GmbH, Heidelberg, Germany) and cultured in RPMI/10% FCS at 37 °C under cell culture conditions. Non-adherent cells were aspirated with the medium after 24 h of cultivation, and cells were cultured for 24 h in the presence or absence of different concentrations of freshly prepared Ag-NP or Ag^+^ ions (silver acetate solution, normalized to the silver content) in a humidified atmosphere of 5% CO_2_.

PVP-coated spherical Ag-NP were dispersed in sterile ultrapure water at 1 mg·mL^−1^ as the stock solution. Dilutions of Ag-NP were also prepared in ultrapure water. The final Ag-NP concentrations were 10 µg·mL^−1^, 5 µg·mL^−1^ and 2.5 µg·mL^−1^, and the final silver ion concentrations were 1.0 µg·mL^−1^, 0.5 µg·mL^−1^ and 0.25 µg·mL^−1^. All of the silver concentrations given here refer to the amount of silver as determined by AAS.

After 24 h of incubation, the particle-containing medium was removed, and the cells were washed and incubated with adipogenic or osteogenic differentiation medium (Life Technologies). After 14 d (adipogenesis) or 21 d (osteogenesis) of cultivation, adipogenic cultures were processed for oil red staining or Bodipy^493/503^ staining and osteogenic cultures were processed for alizarin red staining. To guarantee non-toxic effects during these prolonged incubation periods, lower Ag-NP-concentrations were used compared with the uptake studies (up to 10 µg·mL^−1^).

To support chondrogenic differentiation, 400,000 cells were incubated with different concentrations of Ag-NP/Ag^+^ ions for 24 h and pelleted afterwards. After cultivation for 3 h under cell culture conditions, prewarmed chondrogenic differentiation medium was added. After 21 d of cultivation, chondrogenic pellets were stained by alcian blue. Undifferentiated hMSCs incubated in the presence of RPMI/10% FCS served as the negative control. hMSCs incubated in the presence of the corresponding differentiation medium alone served as the positive control.

In all experiments, hMSCs treated with adipogenic, osteogenic or chondrogenic differentiation medium in the presence of nanoparticles were compared with cells cultured without differentiation medium in the presence of RPMI/10% FCS and Ag-NP/Ag^+^ ions and with cells incubated without Ag-NPs/ Ag^+^ ions in the presence of the differentiation medium alone (100% differentiation).

#### Measurement of cell viability

The viability of the incubated hMSCs was analyzed by using calcein-acetoxymethylester (calcein-AM, Calbiochem, Schwalbach, Germany) fluorescence staining. After incubation for 24 h, the nanoparticle- and silver ion-treated cells were washed, and particle-free medium (RPMI/10% FCS or adipogenic, osteogenic differentiation medium) was added. After 14 d or 21 d, the cells were washed twice with RPMI and incubated with Calcein-AM (1 µM) at 37 °C for 30 min under cell culture conditions. Subsequently, the adherent cells were washed again with RPMI and analyzed by fluorescence microscopy (Olympus MVX10, Olympus, Hamburg, Germany). Fluorescence micrographs were taken (Cell P, Olympus) and digitally processed using Adobe Photoshop^®^ 7.0.

#### Quantitative cell differentiation

Staining with alizarin red S was used to monitor the degree of mineralization of osteogenic differentiated hMSCs in the presence or absence of Ag-NP/silver ions. Briefly, cells were washed with PBS and fixed with 10% formaldehyde for 30 min. After fixation, the cells were washed three times with distilled water and stained with 1% alizarin red S solution (Sigma-Aldrich) for 5 min. The differentiation rates of hMSCs were assessed on an EVOS xl core light microscope (PEQLAB Biotechnologie GMBH, Erlangen, Germany). To quantify the staining, the cells were washed with distilled water and incubated with 1 mL of 10% cetylpyridinium chloride (Sigma-Aldrich) with shaking on a plate rotator. The extracted supernatant was collected, and the optical density was measured at a wavelength of 570 nm by using a Microplate Reader (MRX Revelation, Dynex Technologies). The mineralization rate was expressed as the percentage of cells cultured under osteogenic conditions in the absence of silver.

The adipogenic differentiation rates of hMSCs in the absence or presence of Ag-NP/Ag^+^ ions was determined by staining intracytoplasmic lipids and lipoproteins in the vacuoles of cells using oil red O (Sigma-Aldrich) or Bodipy^493/503^. Briefly, after 14 d of incubation, the cells were washed twice with PBS, fixed with 10% formaldehyde for 10 min and then stained with oil red O solution or Bodipy^493/503^ for 15 min. Subsequently, cells were washed twice with distilled water, and the adipogenic differentiation rates of hMSCs were assessed with an EVOS xl core light microscope (PEQLAB Biotechnologie GMBH, Erlangen, Germany) or by fluorescence microscopy (Olympus BX63, Olympus, Hamburg, Germany). To quantify the oil red O content, hMSCs were washed three times with DPBS to remove background staining, and 4% Nonidet P-40 (Roche, Mannheim, Germany) was added to resolve oil red O. The quantitative analysis of lipid accumulation of oil red O was performed by using a UV–vis spectrophotometer (Ultraspec 3100 pro, Amersham Bioscience, GE Healthcare, Freiburg Germany) at a wavelength of 520 nm to measure the optical density of the extracted oil droplets. For Bodipy^493/503^ fluorescence quantification microphotographs were taken (Olympus CellP, Olympus, Hamburg, Germany) and digitally processed using Adobe Photoshop^®^ 7.0.

Chondrogenic differentiation was analyzed by staining the pellet cultures with alcian blue 8GX (Sigma-Aldrich). After 21 d of incubation, cells were washed twice with DPBS and fixed with 4% formaldehyde solution for 30 min. After fixation, cells were washed with DPBS and stained with 1% alcian blue solution for a further 30 min. Subsequently, pellet cultures were rinsed three times with 0.1 N HCL and additionally with water to neutralize the acidity. Incubation with alcian blue stains the polyanionic glycosaminoglycan chains of proteoglycans in chondrocytes. The chondrogenic differentiation of hMSCs was assessed on an EVOS xl core light microscope (PEQLAB Biotechnologie GMBH).

#### ELISA

The secretion of specific differentiation biomarkers (adiponectin, osteocalcin and aggrecan) by Ag-NP/Ag^+^ ion-treated hMSCs was quantified in the media by using specific enzyme-linked immunosorbent assays (ELISA). The antibodies and the recombinant human protein standards were supplied by R&D Systems (Adiponectin; Wiesbaden, Germany), Life Technologies (Aggrecan; Darmstadt, Germany) and Quidel (Osteocalcin; Berlin, Germany). The biomarkers were quantified using the manufacturer’s ELISA protocols. The ELISA microtiter plates were evaluated on an ELISA reader (MRX Revelation, Dynex Technologies) set to 450 nm.

#### Statistical analysis

Data are expressed as the mean ± SD of at least three independent experiments. Analysis of the data distribution was performed using the Student’s *t*-test to analyze the significance of differences between the treated group and the control group without silver exposure. Significant differences between groups of data were assessed by performing One Way ANOVA followed by the Tukey post-hoc test. Values of *p* of less than 0.05 were considered statistically significant.
